# Infection by Physicians

**Published:** 1890-04

**Authors:** 


ARTICLE VII.

### INFECTION BY PHYSICIANS.

In the *Centralblatt fur Chirurgie* for September 28, 1889, Professor A. Neisser, of Breslau, discusses the question whether the physician who has been infected with syphilis should on that account limit his surgical and obstetrical activity. He cites the cases of four of his assistants who had acquired a finger-infection, and states that Fritsch had had eight assistants and six midwives similarly infected, all of whom continued their work. Other illustrations of infected persons and of transmission of syphilis by them are referred to.

Professor Neisser concludes that it is in the first two or three years of the disease only that there is any danger, and he believes that this danger is much lessened by active treatment.

He warns especially against the infected person doing obstetrical or surgical work with the specific eruption on his hands. A syphilitic person, also, who has a non-luetic eruption on the hand, should be extremely careful; furthermore Neisser inclines to the belief that in the first year or two of syphilis the blood is infective, and that an accidental cut of the surgeon's hand would be dangerous.

However, with suitable precautions, the physician or surgeon who is infected can safely carry on his practice, and need hardly limit his work. The infected midwife is a much more dangerous person, for she usually has not the intelligence or honesty to recognize an affection if she have one.

A much more serious source of danger, in obstetrical practice particularly, is a puerperal fever of severe type. *The Northwestern Lancet* recently, in discussing this subject, recalls the classical case of Dr. Rutter, of Philadelphia. In the year 1843 he had forty five cases of puerperal fever in his practice, and in the next year twenty-five, while the other physicians of Philadelphia saw little or nothing of the disease. No amount of bathing, change of clothing, even shaving the head, had any effect. At last, in desperation, he went away from the city for ten days, and when he approached his next patient wore entirely new clothing and a new wig, leaving even watch and pencil-case at home. Yet this patient, too, was infected in the same way and died. Thirty years afterward, Dr. Rutter's frightful record was explained by the disclosure of the fact that he was suffering at the time from a muco-purulent nasal catarrh, probably an ozæna.

A writer in *The London Lancet* describes two cases coming to his knowledge where medical men infected their puerperal patients through septic discharges from their own persons. In one case the source of infection was a slight but offensive discharge from the middle ear, the sufferer from which had a large midwifery practice with a great deal of septicæmia.

The other case was one of interest in connection with Neisser's article to which we have referred It was that of an assistant who went to attend a case of childbirth in the absence of his principal. The woman died of puerperal fever, and investigation showed that the assistant was afflicted with tertiary syphilis and had an ozæna.

Such cases show us that physicians cannot be too careful of their persons when attending obstetrical as well as surgical cases.—*Medical Record.*

